# Reduced Genetic Diversity of Key Fertility and Vector Competency Related Genes in *Anopheles gambiae* s.l. Across Sub-Saharan Africa

**DOI:** 10.3390/genes16050543

**Published:** 2025-04-30

**Authors:** Fatoumata Seck, Mouhamadou Fadel Diop, Karim Mané, Amadou Diallo, Idrissa Dieng, Moussa Namountougou, Abdoulaye Diabate, Alfred Amambua-Ngwa, Ibrahima Dia, Benoit Sessinou Assogba

**Affiliations:** 1 Medical Research Council Unit, The Gambia at the London School of Hygiene and Tropical Medicine, Banjul P.O. Box 273, The Gambia; seckafatoumata@gmail.com (F.S.); mouhamadou-fadel.diop@lshtm.ac.uk (M.F.D.); karim.mane@lshtm.ac.uk (K.M.); alfred.ngwa@lshtm.ac.uk (A.A.-N.); 2 Institut Pasteur de Dakar, Dakar 220, Senegalidrissa.dieng@pasteur.sn (I.D.); ibrahima.dia@pasteur.sn (I.D.); 3 Institut de Recherche en Sciences de la Santé (IRSS), Bobo-Dioulasso 01 BP 545, Burkina Faso; namountougou_d@yahoo.fr (M.N.); npiediab@gmail.com (A.D.)

**Keywords:** genes, genetic diversity, fertility, vector competency, *Anopheles gambiae* s.l., sub-Saharan Africa

## Abstract

Background: Insecticide resistance challenges the vector control efforts towards malaria elimination and proving the development of complementary tools. Targeting the genes that are involved in mosquito fertility and susceptibility to *Plasmodium* with small molecule inhibitors has been a promising alternative to curb the vector population and drive the transmission down. However, such an approach would require a comprehensive knowledge of the genetic diversity of the targeted genes to ensure the broad efficacy of new tools across the natural vector populations. Methods: Four fertility and parasite susceptibility genes were identified from a systematic review of the literature. The Single Nucleotide Polymorphisms (SNPs) found within the regions spanned by these four genes, genotyped across 2784 wild-caught *Anopheles gambiae* s.l. from 19 sub-Saharan African (SSA) countries, were extracted from the whole genome SNP data of the Ag1000G project (Ag3.0). The population genetic analysis on gene-specific data included the determination of the population structure, estimation of the differentiation level between the populations, evaluation of the linkage between the non-synonymous SNPs (nsSNPs), and a few statistical tests. Results: As potential targets for small molecule inhibitors to reduce malaria transmission, our set of four genes associated with *Anopheles* fertility and their susceptibility to *Plasmodium* comprises the mating-induced stimulator of oogenesis protein (*MISO*, AGAP002620), *Vitellogenin* (*Vg*, AGAP004203), *Lipophorin* (*Lp*, AGAP001826), and *Haem-peroxidase 15* (*HPX15*, AGAP013327). The analyses performed on these potential targets of small inhibitor molecules revealed that the genes are conserved within SSA populations of *An. gambiae* s.l. The overall low Fst values and low clustering of principal component analysis between species indicated low genetic differentiation at all the genes (*MISO, Vg, Lp* and *HPX15*). The low nucleotide diversity (>0.10), negative Tajima’s D values, and heterozygosity analysis provided ecological insights into the purifying selection that acts to remove deleterious mutations, maintaining genetic diversity at low levels within the populations. None of *MISO* nsSNPs were identified in linkage disequilibrium, whereas a few weakly linked nsSNPs with ambiguous haplotyping were detected at other genes. Conclusions: This integrated finding on the genetic features of major malaria vectors’ biological factors across natural populations offer new insights for developing sustainable malaria control tools. These loci were reasonably conserved, allowing for the design of effective targeting with small molecule inhibitors towards controlling vector populations and lowering global malaria transmission.

## 1. Introduction

Caused by *Plasmodium* parasites and transmitted by female *Anopheles* mosquitoes, malaria remains a significant public health concern. According to the latest estimations from the World Health Organization (WHO), malaria caused 597,000 deaths worldwide in 2023, with 97% of those cases occurring in Africa, primarily in sub-Saharan Africa [1].

Between 2000 and 2015, malaria incidence significantly decreased, with an estimated 663 million clinical cases averted. Of these cases, 68% were attributed to the use of insecticide-treated bed nets (ITNs), and 10% to indoor residual spraying (IRS) [2]. This demonstrates the substantial impact of vector control in the programs established to reduce the malaria burden. However, since 2016, malaria cases have been on the rise [1]. The extensive use of massive insecticide-based control interventions has led to the widespread development of insecticide resistance [3,4,5,6,7] and adaptive behavioral changes in vector populations [8,9,10]. This situation poses challenges to malaria elimination and highlights the need for the development of novel vector control tools.

The Sterile Insect Technique (SIT) holds promise for managing various insect pests, including *Anopheles* mosquitoes. However, this technique has limitations, particularly the requirement for efficient sex-separation systems to prevent the release of female mosquitoes and reduce the risk of disease transmission [11]. Additionally, the dispersal and fitness (survival and competitiveness) of released sterile insects are lower compared to wild populations [12]. Alternatively, direct sterilization of female *Anopheles* by disrupting the reproductive synergy with specific gene-targeted small molecule inhibitors could be an innovative approach to control malaria transmission. Molecular pathways underlying *Anopheles* reproductive biology have been extensively studied over the last decade, and the role of certain proteins involved has been established [13,14,15,16,17,18,19,20,21,22,23,24].

For this study, we conducted a brief systematic review on known genes involved either in *Anopheles* fertility or infectivity to *Plasmodium* to identify those associated with both biological factors. Studies have shown that investigating mosquito traits of fertility and immunity is necessary for the detection of potential targets for novel vector control strategies [25]. Four thoroughly characterized genes were selected as prospective targets for small molecule inhibitors: the mating-induced stimulator of oogenesis protein (*MISO*), the nutrients transporters, *Vitellogenin* (*Vg*) and *Lipophorin* (*Lp*), and the *Heme-peroxidase 15* (*HPX15*) (see File S1).

Nevertheless, such an approach requires prior knowledge of the genetic structure of the target genes in natural populations for evidence-based control. The genetic variation at the nucleotide level can lead to changes in phenotype as non-synonymous mutations can influence the protein’s structure, function, or binding properties [26] and are often subject to natural selection.

Advancements in genome sequencing and the accessibility of next-generation sequencing (NGS) technologies are key to malaria surveillance and intervention programs. They provide updated insights into malaria genomic epidemiology, including demographic and evolutionary trends in both mosquitoes [27] and parasite [28] populations, some imposed by interventions. These technologies also offer opportunities to investigate and predict genetic variations underlying important traits, such as insecticide resistance [29,30,31] and drug resistance [32], but have not been adequately explored to assess variation in susceptibility to malaria parasite infections and fertility, two important biological factors that may impact variance in malaria across populations.

Here, we analyzed the genetic variation in selected target genes involved in the fertility and vector competency of the species in the *An. gambiae* complex populations across sub-Saharan African populations. Genetic variants were obtained from whole genomic Single Nucleotide Polymorphisms (SNPs) data of the phase 3 (Ag3.0) of the *An. gambiae* 1000 Genomes project (Ag1000G). The goal was to identify the most conserved genes as suitable targets for vector sterilization and a potential reduction of infectivity to *Plasmodium.*

## 2. Results

### 2.1. Potential Targetable Genes Involved in Anopheles gambiae s.l. Fertility and Plasmodium Infectivity

**Figure 1:**
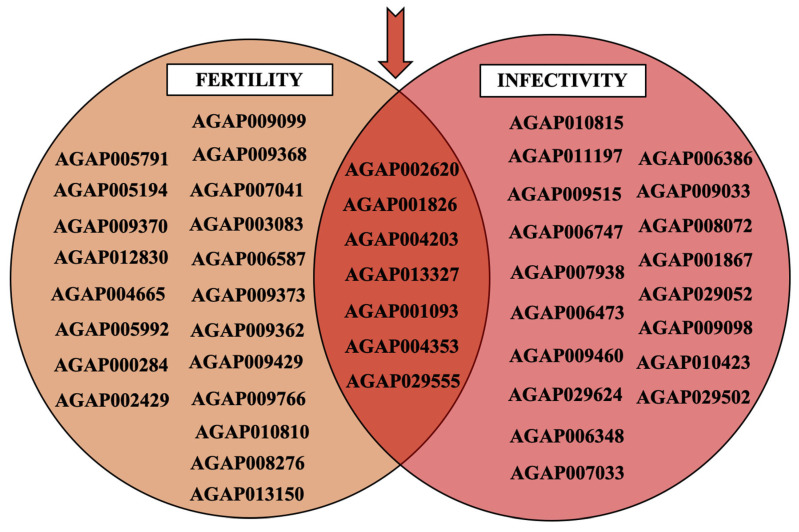
Identified genes involved in the fertility and/or infectivity of *Anopheles gambiae s.l.* This Venn diagram shows the list of genes identified from our literature review. Among the 27 genes identified as involved in *An. gambiae* s.l. mosquito fertility and the 25 genes identified as involved in their infectivity to *Plasmodium*, seven genes have been identified as having a possible implication in both fertility and infectivity (genes in the overlapping region between the two circles). The gene names are referred to the *An. gambiae* s.s. *PEST* gene annotation from Vectorbase.

Many genes express differently for controlling post-mating pathways (e.g., egg production, sperm storage, refraction to subsequent mating) and post-blood-feeding ones (e.g., egg maturation and immune response to pathogens as *Plasmodium*) of mated *Anopheles gambiae* females. The brief systematic review identified 27 genes that are involved in *An. gambiae* s.l. mosquito fertility, and 25 genes in their infectivity to *Plasmodium* (Figure 1). Amongst these, seven genes are associated with both *Anopheles* fertility and infectivity to *Plasmodium* (genes in the overlapping region between the two circles). Amongst these, the *mating-induced stimulator of oogenesis protein* (*MISO, AGAP002620*), the *Heme-peroxidase 15* (*HPX15*, AGAP013327), the *major yolk protein precursor Vitellogenin* (*Vg, AGAP004203*), and the *major vitellogenic lipid transporter Lipophorin* (*Lp, AGAP001826*) were selected as key targetable genes for vector control (see Supplementary Materials Introduction).

### 2.2. Low Population Differentiation in the Four Targetable Genes Involved in Anopheles gambiae s.l. Fertility and Plasmodium Infectivity

**Figure 2:**
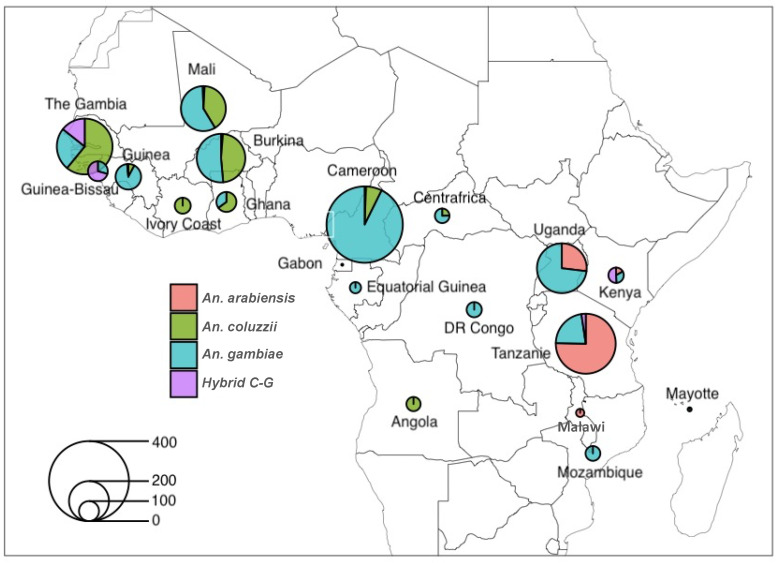
Map of distribution and composition of *Anopheles gambiae* s.l. database used from 19 sub-Saharan African countries. A total of 2784 whole genomes vcf SNP datasets of wild-caught *Anopheles gambiae* s.l. mosquitoes, collected in 19 sub-Saharan African countries, were utilised in this study. These datasets were obtained from the phase 3 Ag1000G project (https://www.malariagen.net/data_package/ag1000g-phase3-snp/, accessed on 6 August 2021). The numbers within the bar plots indicate the sample count for each population or species from a specific country.

**Figure 3:**
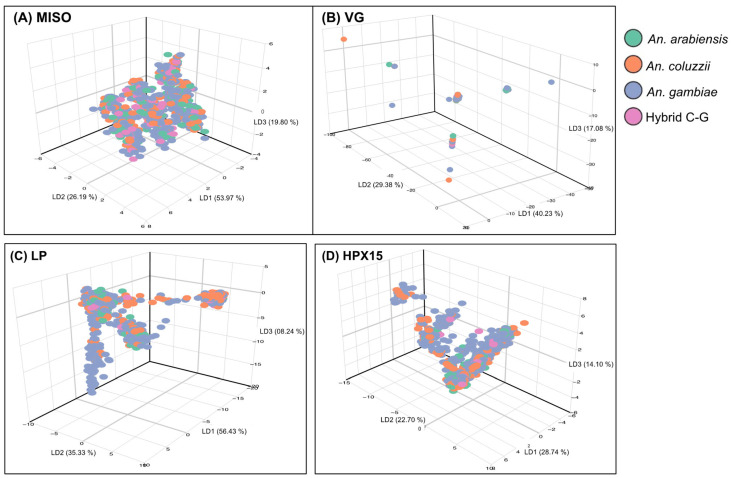
Principal component analysis (PCA) performed on four targetable genes involved in *Anopheles gambiae* s.l. fertility and Plasmodium infectivity using sequencing data from Malariagen phase 3 Ag1000G project (see Figure 2). (**A**) Mating-induced stimulator of oogenesis protein (*MISO*, AGAP002620), (**B**) *Vitellogenin* (*VG*, AGAP004203), (**C**) *Lipophorin* (*LP*, AGAP001826), and (**D**) *Haem-peroxidase 15* (*HPX15*, AGAP013327). Within the *Anopheles gambiae* s.l. species, the PCA showed a lower separation among species based on the components analyzed in this dataset at each gene.

**Figure 4:**
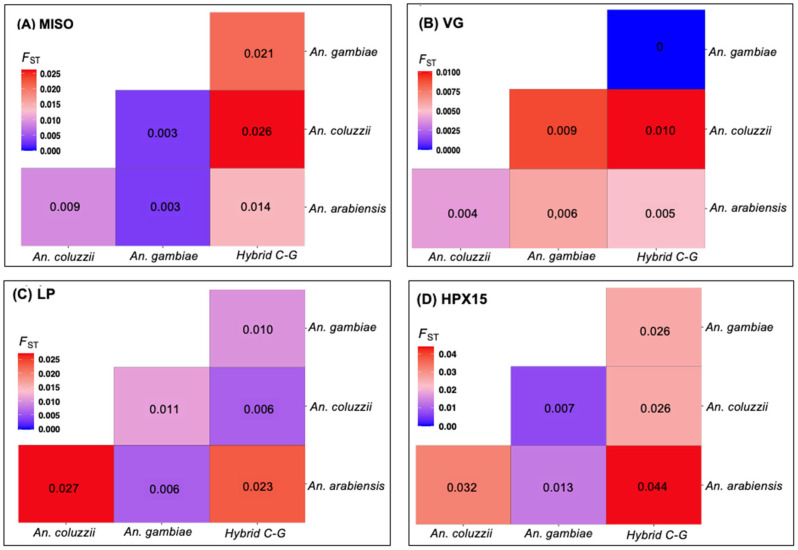
Global pairwise fixation index (*F_ST_*) performed on four targetable genes involved in *Anopheles gambiae* s.l. fertility and *Plasmodium* infectivity using sequencing data from Malariagen phase 3 Ag1000G project (see Figure 2). (**A**) Mating-induced stimulator of oogenesis protein (*MISO*, AGAP002620), (**B**) *Vitellogenin* (*VG*, AGAP004203), (**C**) *Lipophorin* (*LP*, AGAP001826), and (**D**) *Haem-peroxidase 15* (*HPX15*, AGAP013327). For each gene, the overall *F_ST_* between species was generally low, ranging from dark-blue to dark-red.

**Figure 5:**
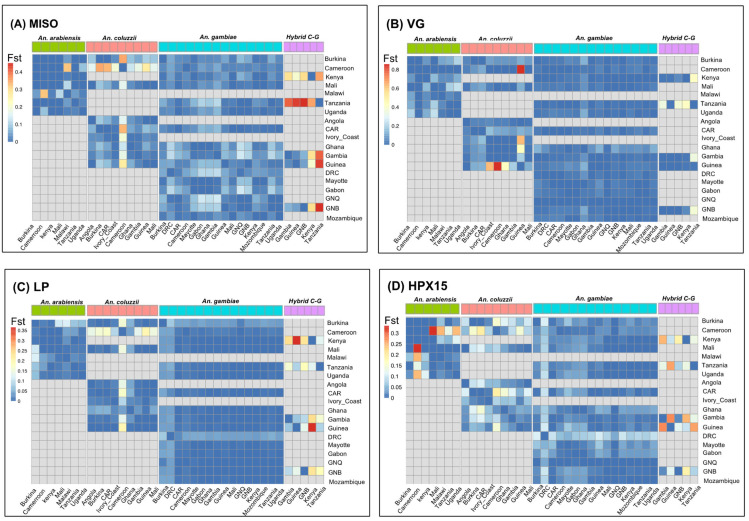
Heatmap of pairwise fixation index (Fst) between populations for each of four species on four targetable genes involved in *Anopheles gambiae* s.l. fertility and *Plasmodium* infectivity using sequencing data from Malariagen phase 3 Ag1000G project. (**A**) Mating-induced stimulator of oogenesis protein (*MISO*, AGAP002620), (**B**) Vitellogenin (*VG*, AGAP004203), (**C**) *Lipophorin* (*LP*, AGAP001826), and (**D**) *Haem-peroxidase 15* (*HPX15*, AGAP013327). Abbreviations: GNB: Guinea Bissau, DRC: Democratic Republic of Congo, CAR: Central African Republic, GNQ: Equatorial Guinea, Hybrid C-G: Hybrid *Anopheles coluzzii-gambiae*. This Fst heatmap showed a low genetic differentiation between populations for *Anopheles arabiensis, Anopheles coluzzii, and Anopheles gambiae* at all the four genes. However, the heatmap also showed a moderate genetic differentiation between populations for hybrid *Anopheles coluzzii-gambiae* at all four genes.

Using SNP data from 2784 wild-caught *Anopheles gambiae* s.l. species collected across 19 sub-Saharan African countries (Figure 2 and Supplementary Materials Figure S2), the principal component analysis (PCA) identified subpopulations between and within species for *MISO*, *Vg*, and *HPX15*, identifying hybrid cluster between species (Figure 3). The PCA analysis indicated less separation amongst species based on the components analyzed in this dataset at each of the genes (Figure 3A–D). The LP gene showed the most significant separation between species cluster (Figure 3C). In contrast, the *MISO* gene showed weaker genetic differentiation with more homogeneous distribution across species (Figure 3A). However, there was an overall low pairwise fixation index (Fst, Fixation Index of genetic differentiation between populations) between species low (0.005–0.051, 0.001–0.146, 0.002–0.022, and 0.006–0.048, for *MISO*, *Vg*, *Lp* and *HPX15*, respectively), with the highest divergence being against *An. coluzzii* populations for *MISO*, *Vg*, *Lp* genes and against *An. arabiensis* for HPX15 gene (Figure 4). For all genes, the global Fst were very low (less than 0.03) between *An. gambiae* s.l. species. Interestingly, the Fst analysis between *An. gambiae* s.l. species (Figure 4) between geographic populations within species reinforced the observed low differentiation (Figure 5). The Fst values were mostly ranged within 0.10–0.15 between populations for *An. arabiensis, An. coluzzii* and *An. gambiae* at all four genes (Figure 5), except for *HPX15 in An. arabiensis* from Cameroon (Figure 5D). Meanwhile, a moderate genetic differentiation between populations was observed within the hybrid *Anopheles coluzzii*-*gambiae* in all four genes, especially against populations from Kenya and Tanzania against those from Guinea Bissau, Guinea and Gambia in West Africa, reaching Fst values of up to 0.6 for *MISO* (Figure 5A).

### 2.3. Low Genetic Diversity of the Selected Targetable Genes

**Figure 6:**
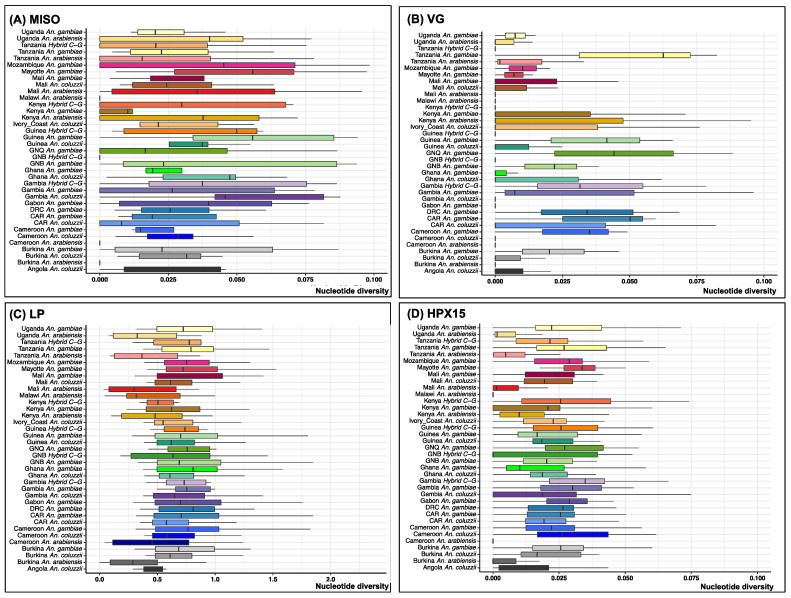
Boxplot of nucleotide diversity (Pi) for each of *Anopheles* species populations on four targetable genes involved in *Anopheles gambiae* s.l. fertility and *Plasmodium* infectivity using sequencing data from Malariagen phase 3 Ag1000G project. (**A**) Mating-induced stimulator of oogenesis protein (*MISO*, AGAP002620), (**B**) *Vitellogenin* (*VG*, AGAP004203), (**C**) *Lipophorin* (*LP*, AGAP001826), and (**D**) *Haem-peroxidase 15* (*HPX15*, AGAP013327). Abbreviations: GNB: Guinea Bissau, DRC: Democratic Republic of Congo, CAR: Central African Republic, GNQ: Equatorial Guinea, Hybrid C-G: Hybrid *Anopheles coluzzii-gambiae*. The nucleotide diversity was overall low (>0.10) in all the population at MISO, VG, LP, and HPX15 genes (see panels (**A**–**D**)).

Genetic diversity for the 2784 wild-caught *Anopheles gambiae* s.l. was estimated by nucleotide diversity (Pi), Tajima’s D selection index, heterozygosity and the linkage disequilibrium (LD) of the non-synonymous SNPs (nsSNPs). Overall, Pi was consistently low in all populations (Figure 6). Most of the median values were less than 0.05 for MISO, VG and HPX15 genes (Figure 6A,B,D), and within 0.5 to 1 for the LP gene (Figure 6C). This low nucleotide diversity suggests potential genetic drift, recent population bottlenecks, or selective sweeps, which are a consequence of positive selection, resulting in the rapid increase in the frequency of one or a limited number of advantageous alleles, reducing the genetic diversity in the populations.

**Figure 7:**
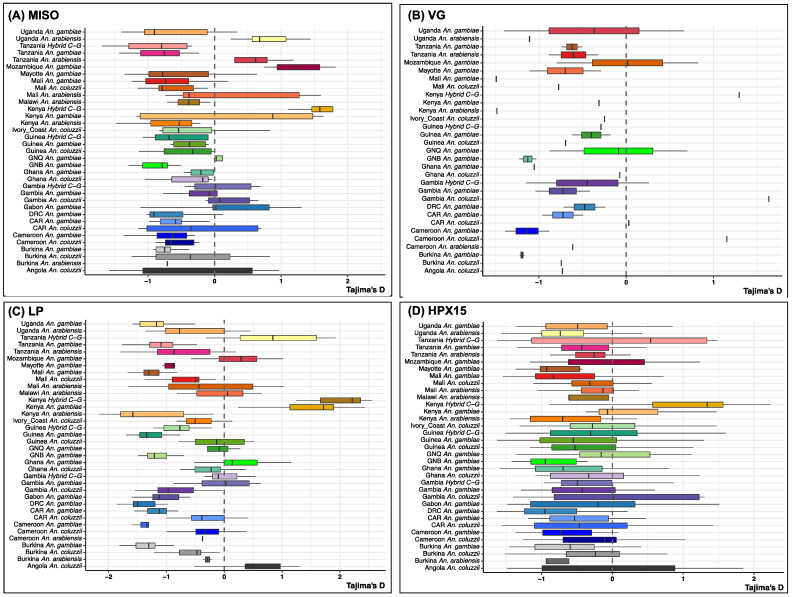
Boxplot of population selection index (Tajima’s D) within each of *Anopheles* species populations on four targetable genes involved in *Anopheles gambiae* s.l. fertility and *Plasmodium* infectivity using sequencing data from Malariagen phase 3 Ag1000G project. (**A**) Mating-induced stimulator of oogenesis protein (*MISO*, AGAP002620), (**B**) *Vitellogenin* (*VG*, AGAP004203), (**C**) *Lipophorin* (*LP*, AGAP001826), and (**D**) *Haem-peroxidase 15* (*HPX15*, AGAP013327). Abbreviations: GNB: Guinea Bissau, DRC: Democratic Republic of Congo, CAR: Central African Republic, GNQ: Equatorial Guinea, Hybrid C-G: Hybrid *Anopheles coluzzii-gambiae*. The Tajima’s D within each of the *Anopheles* species populations on four targetable genes was overall negative, suggesting a purifying selection which eliminates deleterious mutations and leads to a reduction in genetic diversity.

Furthermore, the Tajima’s D values for each target locus within each *Anopheles* species population were mainly negative (Figure 7), suggesting an excess of rare alleles due to population growth of positive selection that reduces genetic diversity. However, there were exceptions of positive Tajima’s D values for some species and populations. For MISO, Tajima D was positive for *An. gambiae* s.s from Mozambique hybrids from Kenya, and *An coluzzii* from Kenya (Figure 7A). Four *An. gambiae* populations (from Mozambique, Kenya, Ghana and Gambia), *An. coluzzii* from Angola, and hybrid C-G (from Kenya and Tanzania) had positive values at the LP gene (Figure 7C). At the *HPX15* gene, positive Tajima’s D was observed only for the hybrid population from Kenya and Tanzania (Figure 7D).

Additionally, the observed heterozygosity and the expected heterozygosity (potential genetic diversity within the population under the assumption of Hardy–Weinberg equilibrium) were estimated for each species population across the four target genes (Supplementary Materials Figure S3). In each case, the heterozygosity values were low, and the observed heterozygosity did not significantly deviate from the expected heterozygosity. These results suggest populations with reduced genetic diversity as observed with Pi and Tajima’s D. Likewise, the pairwise linkage disequilibrium (LD) measurement between non-synonymous SNPs (nsSNPs) across the four genes was weak (Supplementary Materials Figure S4), with no associated SNPs found at the *MISO* gene. Some linked nsSNPs were found in the other genes, especially in the *HPX15*. However, no dominant haplotype (pairs of consecutive SNPs on the same chromosome with high LD, thus inherited together) was detected, with the exception of three SNPs in the *HPX15* gene (between 3L_10786531 and 3L_10786542).

## 3. Discussion

The burden of malaria in Africa remains staggering, with approximately half a million fatalities and two hundred million clinical cases reported annually [1]. Despite the widespread distribution and use of insecticide-treated bed nets, the efficacy of malaria control efforts has increasingly been jeopardized by the emergence of insecticide-resistant *Anopheles* mosquito populations. In light of the pressing need for novel and complementary vector control strategies, a promising approach to reducing malaria transmission involves the direct sterilization of female *Anopheles* mosquitoes. This innovative strategy could be achieved by applying small molecule inhibitors targeting critical genes of reproductive processes, thereby disrupting the reproductive synergy necessary for population maintenance.

Our study focused on several key genes: *MISO*, *Vg*, *Lp*, and *HPX15*, identified as significant contributors to fertility and vector competency in *Anopheles* mosquitoes. To elucidate the genetic patterns associated with these genes, we conducted a comprehensive genetic analysis of these loci in the natural populations of *Anopheles gambiae* s.l. across 19 countries in sub-Saharan Africa, utilizing SNP data from phase 3 of the Ag1000G project (Ag3.0). These data provided key insights into the genetic homogeneity of the four fertility and vector competency-related genes, required for refining the design of small molecule inhibitors against *Anopheles* as an innovative tool to control malaria transmission.

The main finding of this study is the observed genetic conservation in the four selected fertility and vector competency-related genes amongst *An. gambiae* s.l. populations across sub-Saharan Africa. The absence of genetic clustering and a low pairwise fixation index across the four genes (*MISO*, *Vg*, *Lp*, and *HPX15*) indicate a low differentiation between populations, with a slight divergence observed only in the hybrid population for *MISO*, *Lp*, and *HPX15* genes. Additionally, low heterozygosity (He) and nucleotide diversity (Pi), and overall Tajima’s D suggest purifying selection that eliminates deleterious mutations or inbreeding, which reduces genetic diversity. Moreover, the limited ns-SNPs with low LD and no clear haplotype block supports the overall negative Tajima’s D observed. However, there were exceptions, like positive Tajima’s D observed for the hybrid population from Kenya, which could result from positive selection or demographic factors.

Genetic variation serves as the driving force of evolution, enabling organisms to survive and adapt to their environmental conditions [33,34,35]. Species within the complex *An. gambiae*, including the main vectors *An. gambiae*, *An. coluzzii*, and *An. arabiensis*, are crucial in malaria transmission in sub-Saharan Africa. However, *An. arabiensis* populations exhibit slightly distinct plasticity, behavior, and preferences compared to *An. gambiae* and *An. coluzzii* [36,37,38]. They also display unique patterns of seasonal and ecological distributions, such as a greater tolerance to dry environments [38,39,40,41,42,43,44,45]. These geographical contrasts, reproductive behaviors, or environmental adaptive selections may explain the slight genetic distance between the species. Recent genome-wide studies have shown the *An. gambiae* complex as the most polymorphic vector, contributing to its adaptability to insecticides [27,29]. However, we observed genetic diversity conservation across all four genes and *Anopheles* populations in sub-Saharan Africa, emphasizing evolutionary conservation of mechanisms driving fertility and vector competency. These findings could pave the way for the development of innovative tools targeting those genes and all vectors to sustain malaria control efforts in sub-Saharan Africa. These could include targeted interventions aimed at disrupting mosquito reproduction and overcoming the insecticide resistance challenge. Indeed, the reduced genetic diversity of the identified genes suggests that small molecule inhibitors designed to target these specific genes could have broad applicability across diverse *An. gambiae* s.l. populations as is well documented for drug or vaccine [46,47]. This could lead to cost-effective non-insecticide-based vector control tools such as small molecule inhibitors that can block these enzymes and inhibit malaria transmission by a wide range of mosquitoes. Targeting these conserved genes has the advantage of developing the same tools against multiple species across different geographical regions. Moreover, the potential small molecule inhibitors targeting these genes can be integrated into existing vector control measures (LLINs or IRS approaches to optimize the reproductive disruption and transmission blocking strategies [48,49,50]), and to create synergies and enhance the overall efficacy of malaria control programs.

## 4. Conclusions

Our results present rare integrated findings on the biology factors and genetic features of the major malaria vector in natural populations. By revealing this significant degree of conservation of the genes (*MISO*, *Vg*, *Lp* and *HPX15*) within the members of the complex *An. gambiae* throughout sub-Saharan Africa, our data offer new insights for the development of sustainable malaria control tools. These genes could serve as effective targets for managing vector populations and reducing global malaria transmission. However, the genetics of these conserved genes should not be interpreted as a static condition. Continuous monitoring of mosquito populations is essential to detect any evolving genetic changes or the potential development of resistance against small molecule inhibitors.

## 5. Methods

### 5.1. Selection of Potential Targetable Genes Involved in Anopheles gambiae s.s PEST Fertility and Plasmodium Infectivity

An initial exploratory literature review was conducted to identify genes that are associated with either the *An. gambiae* fertility [13,14,15,16,17,18,19,20,21,22,24,25,51,52,53,54,55,56,57,58,59,60,61,62] or its infectivity to *Plasmodium* [15,21,22,23,58,59,60,61,62,63,64,65,66,67,68,69,70,71,72,73,74,75,76,77,78] (see Supplementary Materials Introduction and Table S1 for more details). Amongst these genes, we selected the set of genes that are involved in both the fertility and infectivity to *Plasmodium* for a further review. At the end of the review process, four genes were retained based on the following criteria that have been well characterized with publication evidence (Supplementary Materials Introduction). Those four genes were annotated based on the VectorBase [62] and named according to the *An. gambiae s.s* PEST genes IDs.

### 5.2. SNP Data Used

We used Single Nucleotide Polymorphisms (SNPs) data from the phase 3 *Anopheles gambiae s.l.* 1000 genomes (Ag1000G) project (Ag3.0), accessible online at the MalariaGEN website (https://malariagen.github.io/vector-data/ag3/download.html#snp-calls-vcf-format, accessed on 6 August 2021) [79]. These SNPs were genotyped from 2784 wild-caught *Anopheles* mosquitoes (including *An. arabiensis*, *An. gambiae* s.s, *An. coluzzii* and hybrids *An. gambiae-coluzzii*) that were collected from 19 sub-Saharan Africa countries (Supplementary Materials Introduction). They were used to investigate the genetic diversity of the selected genes. These data was released in February 2021 on the mosquito data-sharing home page of the Malaria Genomic Epidemiology Network (MalariaGEN) [80].

*An. gambiae* s.s was the predominant and widely dispersed species across sub-Saharan Africa, representing 57% of the dataset. *An. coluzzii* was collected mainly in West Africa (24%), where the highest rate of hybrid *An. gambiae-coluzzii* was observed (6%). *An. arabiensis* was primarily collected in East Africa, accounting for 13% of the dataset (Supplementary Materials Figure S2). 

A comprehensive description of the population sampling, sequencing, alignment, genotyping, quality control, variant calling and filtering methods are available on the Ag3.0 MalariaGEN vector data user guide (https://malariagen.github.io/vector-data/ag3/methods.html, accessed on 6 August 2021). Briefly, mosquito samples were sequenced at high coverage using Illumina technology at the Welcome Sanger Institute, and the MalariaGEN Resource Centre Team conducted a quality control check and preliminary analysis generating the VCF SNPs data as previously described [79].

The whole genome VCF files, metadata samples and other relevant data were downloaded and stored in the high-performance computing server (HPC) at the Medical Research Council Unit of The Gambia’s (MRCG) at LSHTM for the subsequent analysis. Using the *Anopheles gambiae* s.s reference genome annotations, AgamP4 PEST [81] from VectorBase variant data for the four target genes of interest were extracted from the whole genome data using bcftools v1.9 [82]. The data were filtered with vcftools v0.1.17 [83] to exclude low-quality SNPs, retaining only sites with less than 10% genotype missing across individuals, a Phred score above 30, and a read depth beyond 10 but not exceeding 50 using PLINK v1.9 [84]. Finally, SNPs with a minor allele frequency greater than 1% were retained (Supplementary Materials Figure S5).

### 5.3. Data Analysis

**Population structure analysis:** We measured the genetic variance due to genetic structure in populations based on the Wright (1951) fixation index (Fst) [85]. Fst values were estimated between species and populations following the WC84 method (Weir & Cockerham, 1984) [86] implemented in the R package adegenet v2.1.7 [87]. The same R package was also used to perform a principal component analysis (PCA) to identify the main axes of variation in the multivariate dataset as described by Jolliffe [88]. To reduce bias in the Fst and PCA analyses, we pruned out SNPs with pairwise linkage disequilibrium (LD) values r2 > 0.25 within a 50 bp window in each gene. This pruning was done with a step size of 5. After this pruning step, the remaining SNPs located on the shared chromosomes between the species were used to estimate the observed and expected heterozygosity using the R package hierfstat [89].

**Genetic diversity analysis:** To assess the level of polymorphism between and within populations, we calculated the nucleotide diversity (Pi) at each locus using the R package PopGenome [90]. The Pi was defined as the average number of nucleotide differences per site between two sequences [91]. Furthermore, we determined Tajima’s D index per gene, a neutrality derived from comparing the mean number of segregating-sites and the average pairwise differences [92]. Tajima’s D allows for detecting evolutionary departure from neutrality as proposed by Kimura [93] that may be due to selective pressure within populations. Also, as the linkage disequilibrium (LD) could provide highlights on the history of natural selection, gene conversion, mutation, and other forces that cause gene-frequency evolution [94]. We used the R packages LDheatmap v.1.0-6 [95] and Gaston v.1.5.7 to compute pairwise linkage disequilibrium (LD) between different non-synonymous SNP (nsSNPs) sites across all species using the genotype correlation coefficient (r2) index between alleles at physically separate loci. Assessing LD between nsSNPs is valuable for identifying genomic regions that may harbor functionally significant genetic variations.

The observed pairwise LD (r2) was averaged for each inter-SNP distance, and r2 measures for each gene were calculated between all pairs of SNPs within each population.

Data and codes used to analyze the data are available in the GitHub 3.4.9 repository: https://github.com/mfdiop/Genetic_Diversity_Anopheles_Gambiae, accessed on 6 August 2021.

## Data Availability

The original contributions presented in this study are included in the article/Supplementary Materials. Further inquiries can be directed to the corresponding author. Raw sequence data are available on MalariaGEN (https://www.malariagen.net/data_package/ag1000g-phase3-snp/, accessed on 6 August 2021).

## Supplementary Materials

The following supporting information can be downloaded at: https://www.mdpi.com/article/10.3390/genes16050543/s1, SI1 Introduction: Genes involved in fertility and vector competency of *Anopheles gambiae.* Figure S2. Composition of *Anopheles gambiae s.l.* database used. A total of 2784 whole genomes vcf SNP datasets of wild-caught *Anopheles gambiae* s.l. mosquitoes, collected in 19 sub-Saharan African countries, were utilized in this study. These datasets were obtained from the phase 3 Ag1000G project (https://www.malariagen.net/data_package/ag1000g-phase3-snp/, accessed on 6 August 2021). The numbers within the bar plots indicate the sample count for each population or species from a specific country. Figure S3. Observed Heterozygosity (Ho in Blue) and Expected Heterozygosity (He in Gray) for each species on the four targetable genes involved in *Anopheles gambiae s.l.* fertility and *Plasmodium* infectivity using sequencing data from the MalariaGEN phase 3 Ag1000G project. (A) Mating-induced stimulator of oogenesis protein (MISO, AGAP002620), (B) Vitellogenin (VG, AGAP004203), (C) Lipophorin (LP, AGAP001826), and (D) Haem-peroxidase 15 (HPX15, AGAP013327). At each of the genes, the observed heterozygosity was not significantly deviated from the expected heterozygosity providing insights into the low genetic diversity within the populations. Figure S4. Pairwise linkage disequilibrium (LD) measurement between non-synonymous SNPs across the four targetable genes involved in *Anopheles gambiae s.l.* fertility and *Plasmodium* infectivity using sequencing data from the MalariaGEN phase 3 Ag1000G project. (A) Mating-induced stimulator of oogenesis protein (MISO, AGAP002620), (B) Vitellogenin (VG, AGAP004203), (C) Lipophorin (LP/AGAP001826), and (D) Haem-peroxidase 15 (HPX15, AGAP013327). No associated non-synonymous SNPs (nsSNPs) where found at the MISO gene, whereas few and low linked nsSNPs with no clear haplotype block were found in the other genes except the potential haplotype on the HPX15 gene between 3L_10786531 and 3L_10786542 that might be taken into consideration for further investigation. Figure S5. Global and intra-species minor allele frequencies (MAF) distribution of SNPs at four targetable genes involved in *Anopheles gambiae s.l.* fertility and *Plasmodium* infectivity using sequencing data from the MalariaGEN phase 3 Ag1000G project. (A) Mating-induced stimulator of oogenesis protein (MISO, AGAP002620), (B) Vitellogenin (VG, AGAP004203), (C) Lipophorin (LP/AGAP001826), and (D) Haem-peroxidase 15 (HPX15, AGAP013327). The MAF has shown a skewed distribution with most SNPs having low MAF (close to 0) indicating that most SNPs are monomorphic (only one allele is common) in the population, indicating low genetic diversity. Table S1. List of genes reviewed.
