# Supplementary material for: Reduced Genetic Diversity of Key Fertility and Vector Competency Related Genes in Anopheles gambiae s.l. Across Sub-Saharan Africa
Source: Genes (Basel). 2025 Apr 30;16(5):543. doi: 10.3390/genes16050543 (PMC12111087; doi:10.3390/genes16050543)

Supporting Information

SI1: Systematic review on the Selection of potential targetable genes involved in *Anopheles gambiae* fertility and *Plasmodium* infectivity

Systematic review process

To systematically identify potential targetable genes involved in *Anopheles gambiae* fertility and *Plasmodium* infectivity, we conducted an extensive literature search using databases such as PubMed, Web of Science, Scopus, Google Scholar, and VectorBase. We employed Boolean search terms: (1) ("Anopheles gambiae" OR "Anopheles mosquitoes") AND ("fertility" OR "fecundity" OR "reproduction" OR "oogenesis"), (2) ("Anopheles gambiae" OR "Anopheles mosquitoes") AND ("Plasmodium" OR "malaria transmission" OR "vector competence") and (3) ("gene expression" OR "genetic regulation" OR "functional analysis") to extract relevant studies published from 2000 onwards, focusing on genes functionally characterised in either reproductive success or malaria transmission. Genes were categorised into fertility-related (Genes with a direct role in mating, egg development, or reproductive success) and infectivity-related (Genes involved in *Plasmodium* survival, immune evasion, or vector competence), and those overlapping both functions. Candidate genes were selected based on functional characterisation, experimental validation (RNAi, CRISPR, transcriptomics), annotation in VectorBase, and reproducibility across multiple studies. The selection process involved extracting data on gene expression, functional impact, and role in *Plasmodium* infectivity. Only genes with strong experimental evidence were retained. Four genes were selected and annotated based on VectorBase gene IDs at the end of the review process. Studies focused on non-*Anopheles gambiae* species or review papers without primary experimental data were excluded.

Genes involved in fertility and vector competency of *Anopheles gambiae*

The Mating-induced stimulator of oogenesis protein (MISO): *Anopheles* mosquitoes' mating induces *MISO* expression in female atrium, regulated by ecdysone receptor 20E (the male hormone transferred to females during mating). This increased *MISO* expression enhances oogenesis and egg laying[1]. It works in concert with the major vitellogenic lipid transporter Lipophorin, which facilitates oocyte development by accumulating lipids in the ovaries [2]. Additionally, *MISO* shield females against

infection-related fecundity costs [3]. However, it's worth noting that gene silencing of *MISO impairs 20E responsive genes such as Vitellogenin and Lipophorin* [2] and does not affect malaria parasite development.

The Nutrients transporters, Vitellogenin (Vg) & Lipophorin (Lp): Vitellogenin (*Vg*) and Lipophorin (*Lp*) are the two most abundant proteins secreted in *Anopheles* hemolymph [4]. *Vg* is induced by 20-Ecdysone in response to mating and a blood meal [1]. It encodes the yolk protein vitellin that is subsequently transported into egg cells in the ovaries to provide the necessary nutrients for embryonic development, vitellogenesis [2,5]. The major lipid transporter Lp play a crucial role in *Anopheles* egg development (oogenesis) [1]. Lp is constitutively expressed in the hemolymph (with an increased expression level after blood feeding) [4,6] to deliver lipids and fatty acids to energy-consuming tissues such as imaginal discs in larvae and muscles, and ovaries in adult females[7]. Knockdown experiments of *Lp* and *Vg*, respectively, resulted in blocked oogenesis and reduced fecundity in *An. gambiae* females [4]. Moreover, studies have shown the implication of *Lp* and *Vg* in *Plasmodium* survival by promoting development [4,7–10] . The absence of *Lp* or *Vg* enhances the efficiency of the antiparasitic factor Thioester-containing protein TEP1[4].

The Heme-peroxidase 15 (*HPX15*): HPX15 is a protein whose expression dependent on the concentration of 20-Ecdysone, induced by mating in the spermatheca and by a blood meal in the midgut [11]. In the spermatheca, *HPX15* plays a crucial role in preventing oxidative damage of the sperm, which is essential for the fertility of female *Anopheles gambiae* [11]. In the midgut, *HPX15* creates a low immunity zone that favors the development of *Plasmodium* [12]. Interestingly, silencing *HPX15* has been shown to restore midgut anti-plasmodial immunity [12–14].

References

1. Rogers DW, Whitten MMA, Thailayil J, Soichot J, Levashina EA, Catteruccia F. Molecular and cellular components of the mating machinery in Anopheles gambiae females. Proc Natl Acad Sci U S A. 2008;105: 19390–19395. doi:10.1073/pnas.0809723105

2. Baldini F, Gabrieli P, South A, Valim C, Mancini F, Catteruccia F. The interaction between a sexually transferred steroid hormone and a female protein regulates oogenesis in the malaria mosquito Anopheles gambiae. PLoS Biol. 2013;11: e1001695. doi:10.1371/journal.pbio.1001695

3. Marcenac P, Shaw WR, Kakani EG, Mitchell SN, South A, Werling K, et al. A mating-induced reproductive gene promotes Anopheles tolerance to Plasmodium falciparum infection. PLoS Pathog. 2020;16: e1008908. doi:10.1371/journal.ppat.1008908

4. Rono MK, Whitten MMA, Oulad-Abdelghani M, Levashina EA, Marois E. The major yolk protein vitellogenin interferes with the anti-plasmodium response in the malaria mosquito Anopheles gambiae. PLoS Biol. 2010;8: e1000434. doi:10.1371/journal.pbio.1000434

5. Thailayil J, Gabrieli P, Caputo B, Bascuñán P, South A, Diabate A, et al. Analysis of natural female post-mating responses of Anopheles gambiae and Anopheles coluzzii unravels similarities and differences in their reproductive ecology. Sci Rep. 2018;8: 6594. doi:10.1038/s41598-018-24923-w

6. Green EI, Jaouen E, Klug D, Proveti Olmo R, Gautier A, Blandin S, et al. A population modification gene drive targeting both Saglin and Lipophorin impairs Plasmodium transmission in Anopheles mosquitoes. eLife. 2023;12: e93142. doi:10.7554/eLife.93142

7. Atella GC, Silva-Neto MAC, Golodne DM, Arefin S, Shahabuddin M. Anopheles gambiae lipophorin: characterization and role in lipid transport to developing oocyte. Insect Biochem Mol Biol. 2006;36: 375–386. doi:10.1016/j.ibmb.2006.01.019

8. Vlachou D, Schlegelmilch T, Christophides GK, Kafatos FC. Functional genomic analysis of midgut epithelial responses in Anopheles during Plasmodium invasion. Curr Biol CB. 2005;15: 1185–1195. doi:10.1016/j.cub.2005.06.044

9. Mendes AM, Schlegelmilch T, Cohuet A, Awono-Ambene P, De Iorio M, Fontenille D, et al. Conserved mosquito/parasite interactions affect development of Plasmodium falciparum in Africa. PLoS Pathog. 2008;4: e1000069. doi:10.1371/journal.ppat.1000069

10. Upton LM, Povelones M, Christophides GK. Anopheles gambiae blood feeding initiates an anticipatory defense response to Plasmodium berghei. J Innate Immun. 2015;7: 74–86. doi:10.1159/000365331

11. Shaw WR, Teodori E, Mitchell SN, Baldini F, Gabrieli P, Rogers DW, et al. Mating activates the heme peroxidase HPX15 in the sperm storage organ to ensure fertility in Anopheles gambiae. Proc Natl Acad Sci U S A. 2014;111: 5854–5859. doi:10.1073/pnas.1401715111

12. Kumar S, Molina-Cruz A, Gupta L, Rodrigues J, Barillas-Mury C. A Peroxidase/Dual Oxidase System Modulates Midgut Epithelial Immunity in Anopheles gambiae. Science. 2010;327: 1644–1648. doi:10.1126/science.1184008

13. Kajla M, Choudhury TP, Kakani P, Gupta K, Dhawan R, Gupta L, et al. Silencing of Anopheles stephensi Heme Peroxidase HPX15 Activates Diverse Immune

Pathways to Regulate the Growth of Midgut Bacteria. Front Microbiol. 2016;7: 1351. doi:10.3389/fmicb.2016.01351

14. Kajla M, Kakani P, Choudhury TP, Kumar V, Gupta K, Dhawan R, et al. Anopheles stephensi Heme Peroxidase HPX15 Suppresses Midgut Immunity to Support Plasmodium Development. Front Immunol. 2017;8: 249. doi:10.3389/fimmu.2017.00249

SI2 Fig. Composition of *Anopheles gambiae s.l.* database used. A total of 2784 whole genomes vcf SNP datasets of wild-caught *Anopheles gambiae s.l.* mosquitoes, collected in 19 sub-Saharan African countries, were utilized in this study. These datasets were obtained from the phase 3 Ag1000G project (https://www.malariagen.net/data/ag1000g-phase3-snp).The numbers within the bar plots indicate the sample count for each population or species from a specific country.

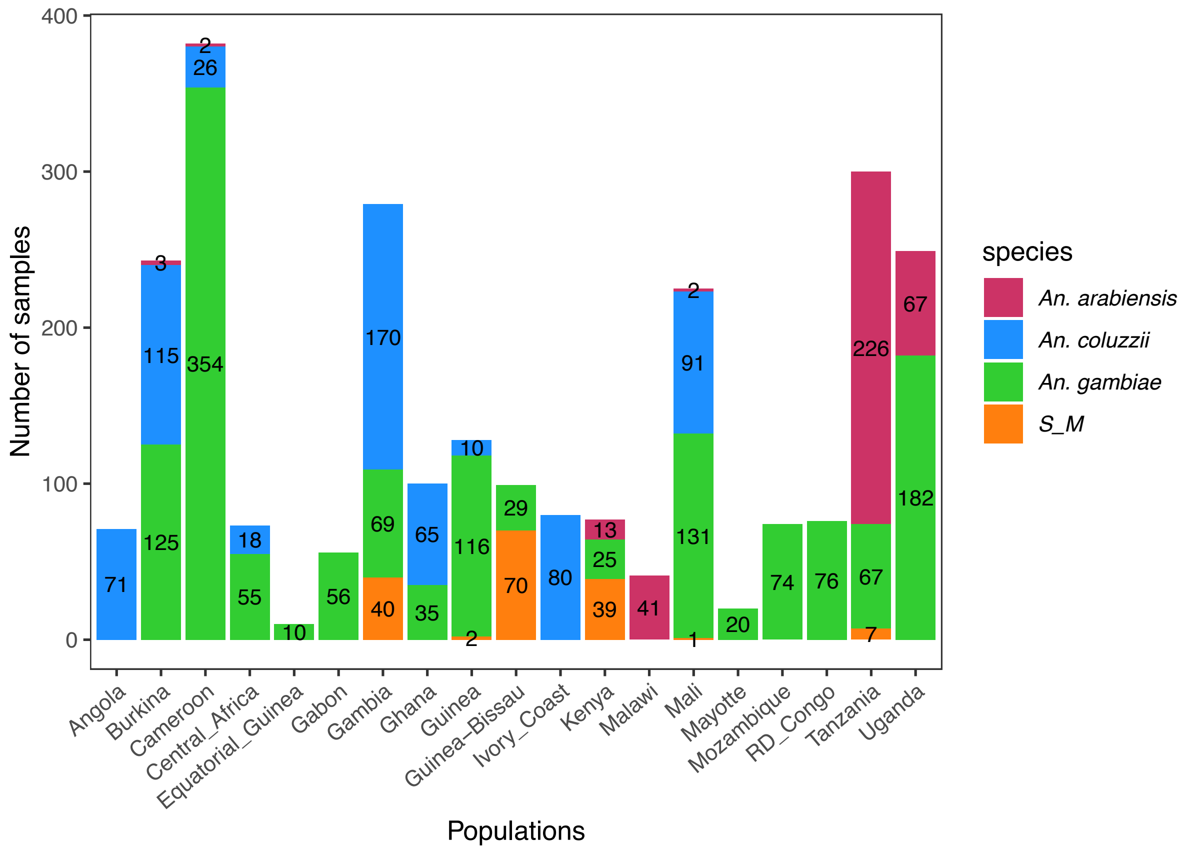

SI3 Fig. Observed Heterozygosity (Ho in Blue) and Expected Heterozygosity (He in Gray) for each species on the four targetable genes involved in *Anopheles gambiae s.l.* fertility and *Plasmodium* infectivity using sequencing data from Malariagen phase 3 Ag1000G project. **A)** Mating induced stimulator of oogenesis protein (MISO, AGAP002620), **B)** Vitellogenin (VG, AGAP004203), **C)** Lipophorin (LP, AGAP001826), and **D)** Haem-peroxidase 15 (HPX15, AGAP013327). At each of the genes, the Observed heterozygosity was not significantly deviated from the expected heterozygosity providing insights into the low genetic diversity within the populations.

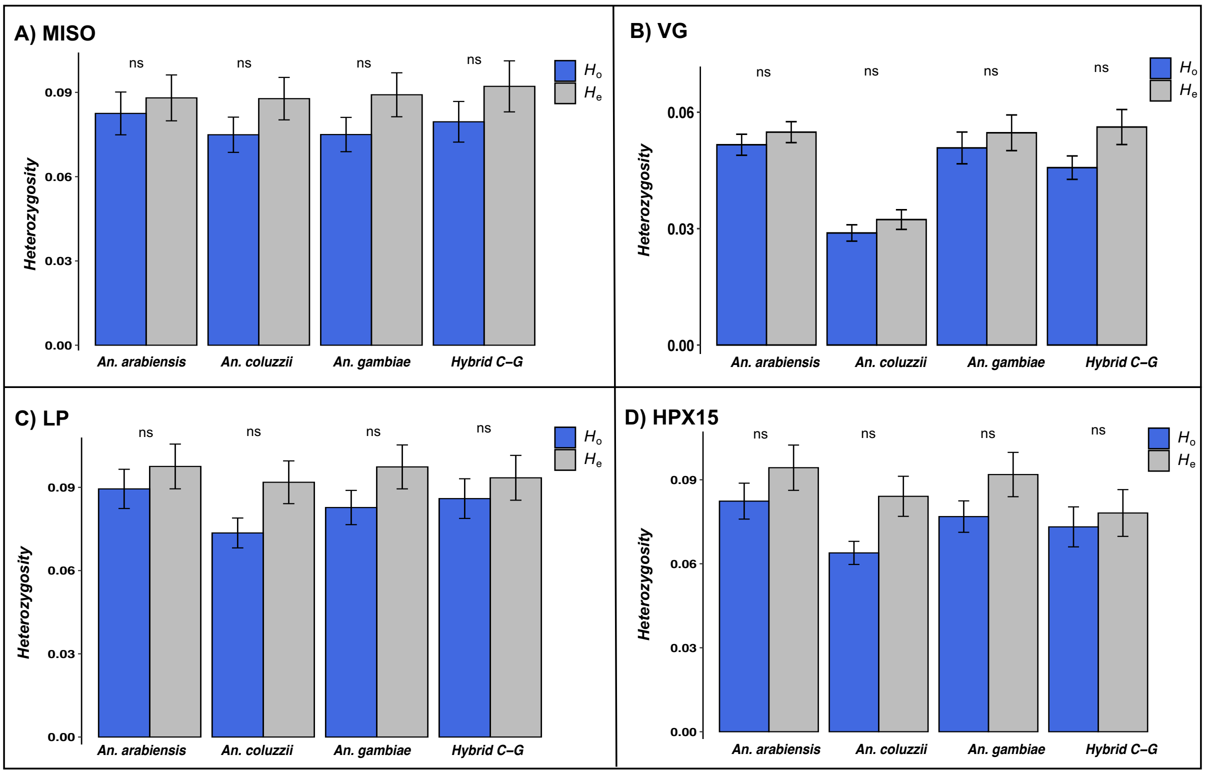

SI4 Fig. Pairwise limkage disequilibrium (LD) measeurement between non synonymous SNPs across the four targetable genes involved in *Anopheles gambiae s.l.* fertility and *Plasmodium* infectivity using sequencing data from Malariagen phase 3 Ag1000G project. **A)** Mating induced stimulator of oogenesis protein (MISO, AGAP002620), **B)** Vitellogenin (VG, AGAP004203), **C)** Lipophorin (LP/AGAP001826), and **D)** Haem-peroxidase 15 (HPX15, AGAP013327). No associated non-synonymous SNPs (nsSNPs) where found at the MISO gene, whereas few and low linked nsSNPs with no clear haplotype block were found in the other genes except the potential haplotype on the HPX15 gene between 3L_10786531 and 3L_10786542 that might be taken into consideration for further investigation

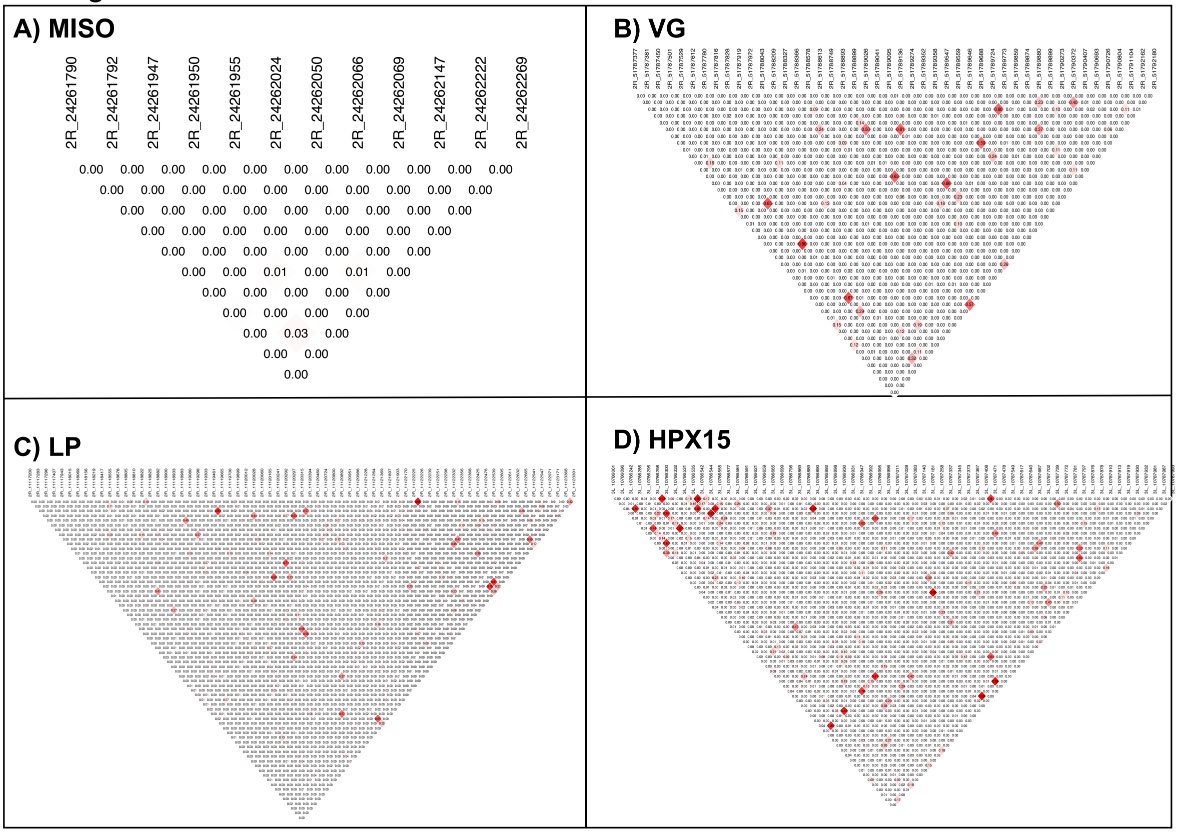

SI5 Fig. Global and intra-species minor allele frequencies (MAF) distribution of SNPs at four targetable genes involved in *Anopheles gambiae s.l.* fertility and *Plasmodium* infectivity using sequencing data from Malariagen phase 3 Ag1000G project. **A)** Mating induced stimulator of oogenesis protein (MISO, AGAP002620), **B)** Vitellogenin (VG, AGAP004203), **C)** Lipophorin (LP/AGAP001826), and **D)** Haem-peroxidase 15 (HPX15, AGAP013327). The MAF has shown a skewed distribution with most SNPs having low MAF (close to 0) indicates that the majority of SNPs are monomorphic (only one allele is common) in the population, indicating a low genetic diversity.

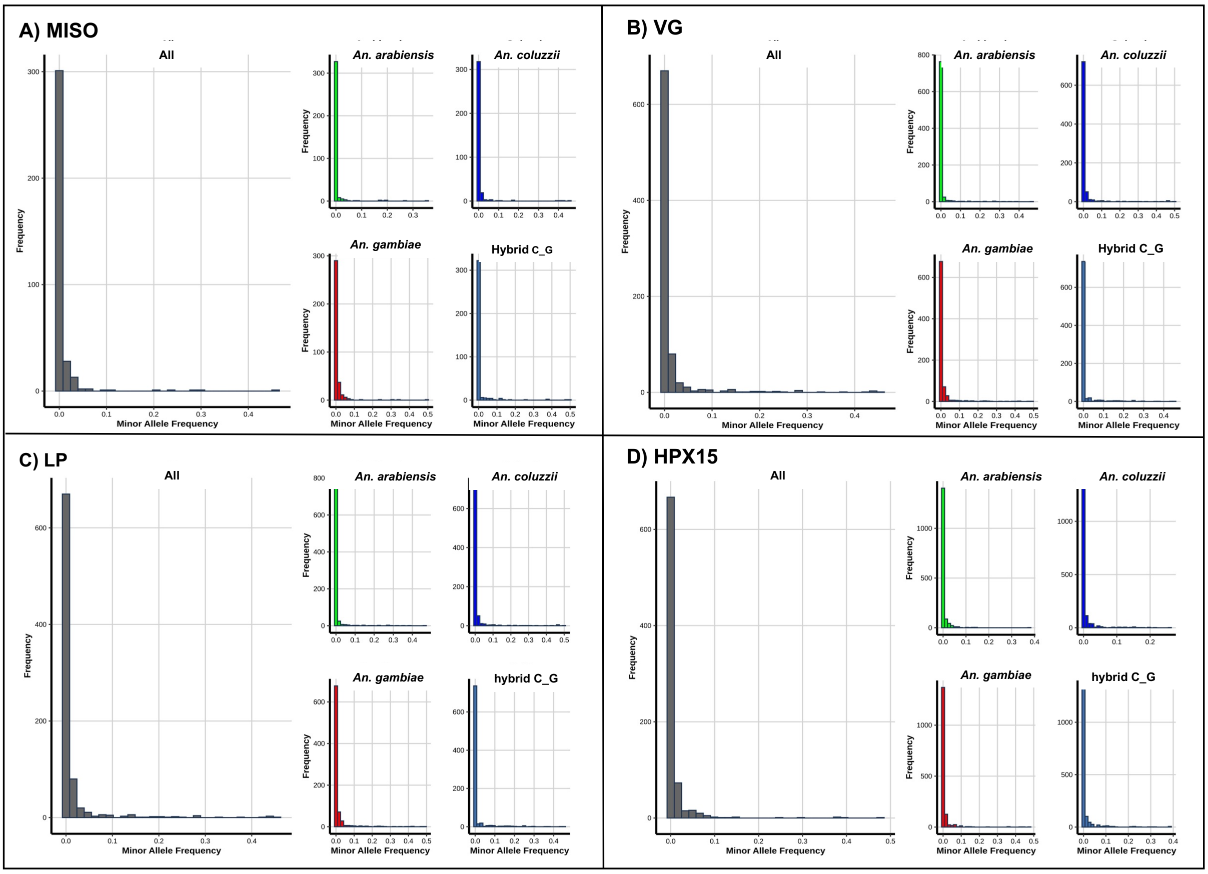

Supplement: Supplementary file 1 [file genes-16-00543-s001.zip › genes-3515486-supplementary/genes-3515486-supplementaryS1toS5.pdf]
